# Newcastle disease virus exploits the phospholipid flippase ATP11c–CDC50A complex to promote viral infection

**DOI:** 10.1016/j.jbc.2025.110584

**Published:** 2025-08-12

**Authors:** Dandan Zhang, Yuechi Hou, Xusheng Qiu, Yang Qu, Yingjie Sun, Cuiping Song, Ying Liao, Chan Ding, Lei Tan

**Affiliations:** 1 Shanghai Veterinary Research Institute, Chinese Academy of Agricultural Sciences, Shanghai, China; 2 College of Veterinary Medicine, South China Agricultural University, Guangzhou, China; 3 School of Agriculture and Biology, Shanghai Jiao Tong University, Shanghai Key Laboratory of Veterinary Biotechnology, Key Laboratory of Urban Agriculture (South), Ministry of Agriculture, Shanghai, China

**Keywords:** Newcastle disease virus, phosphatidylserine, flippase, ATP11c–CDC50A complex, membrane asymmetry, viral pathogenesis

## Abstract

Newcastle disease virus (NDV), a highly contagious avian pathogen, causes significant economic losses in the poultry industry. As an enveloped, negative-sense single-stranded RNA virus, NDV infection and replication are intricately linked to host lipid metabolism, particularly phospholipids like phosphatidylserine (PS). PS asymmetry across the plasma membrane, maintained by phospholipid flippases, such as the ATP11c–CDC50A complex, is crucial for cellular homeostasis but can be exploited by viruses. However, the specific roles of PS and its dynamic regulation by flippases during NDV infection remain unclear. In this study, we investigated how NDV utilizes host PS and the ATP11c–CDC50A complex to facilitate its life cycle. We found that the phospholipid flippase ATP11c–CDC50A complex maintains membrane asymmetry by translocating PS to the inner leaflet, whereas NDV subverts this process by hijacking envelope-associated PS for viral entry and budding. CRISPR–Cas9-mediated ATP11c KO reduced PS flipping efficiency, impaired NDV replication, and disrupted progeny virion release. Notably, CDC50A mutations (D193G/K319E) compromised ATP11c activity, reducing PS redistribution by 60% (*p* < 0.05), highlighting its essential role in flippase function. Mechanistically, NDV-induced apoptosis triggered PS externalization, which enhanced matrix (M) protein clustering at PS-rich membrane domains, significantly increased virus-like particle production (*p* < 0.05). The results reveal that NDV exploits host ATP11c–CDC50A-mediated maintenance of inner-leaflet PS asymmetry to anchor M protein oligomerization at the plasma membrane during replication. These findings fundamentally advance our understanding of viral pathogenesis by elucidating how NDV subverts host lipid homeostasis to fuel its replication cycle.

Newcastle disease, incited by the Newcastle disease virus (NDV), persists as a formidable challenge to the global poultry sector, inflicting substantial economic damage (1). NDV, classified within the *Orthoavulavirus* genus of the Paramyxoviridae family, exhibits a broad avian host range, causing diverse pathologies, including severe respiratory, gastrointestinal, and neurological signs, often culminating in high morbidity and mortality. Its significant economic repercussions and potential for rapid epizootic spread mandate its status as a notifiable disease by the World Organization for Animal Health.

Structurally, NDV is an enveloped virion encapsulating a nonsegmented, negative-sense single-stranded RNA genome (∼15.2 kb). This genome orchestrates the expression of six structural proteins—nucleocapsid (nucleoprotein [NP]), phosphoprotein (P), matrix (M), fusion (F), hemagglutinin–neuraminidase, and the large polymerase (L)—alongside nonstructural proteins, V and W, derived from P gene editing. Viral entry commences with hemagglutinin–neuraminidase attachment to sialic acid–bearing receptors on the host cell surface, followed by F protein–driven fusion of the viral envelope with the host plasma membrane (PM), thereby releasing the viral core into the cytoplasm (2). Subsequent replication and protein synthesis culminate in the assembly of viral components at the PM, a process largely orchestrated by the M protein, leading to the budding and release of progeny virions (3).

Increasingly, the host cell membrane is understood not as a passive barrier, but as an active participant, profoundly influencing viral infection cycles (4). The membrane's lipid composition and architecture, particularly the tightly regulated asymmetric distribution of phospholipids across the bilayer, are crucial determinants of membrane fluidity, curvature, protein function, and cellular signaling (5, 6). Among these lipids, the anionic glycerophospholipid phosphatidylserine (PS), normally constituting 3% to 10% of total cellular lipids, is predominantly sequestered to the inner leaflet of the PM in healthy eukaryotic cells (7). This spatial asymmetry is vital for fundamental cellular processes, including the regulation of membrane protein activity, membrane trafficking, and the orderly execution of apoptosis (8).

The establishment and maintenance of PS asymmetry represent a dynamic equilibrium governed by specialized transmembrane lipid transporters (9). PS translocation and asymmetric distribution at the PM rely on the coordinated activities of ATP-dependent flippases (P4-ATPases), notably ATP11A and ATP11C, which internalize PS; ATP-dependent floppases (ABC transporters) that externalize sphingomyelin and phosphatidylcholine; ATP-independent scramblases that collapse asymmetry upon activation (10). The human genome encodes 14 members of the P4-ATPase family that are evolutionarily conserved across species. Flippases, especially ATP11c at the PM, actively transport phospholipids like PS from the outer (exoplasmic) to the inner (cytoplasmic) leaflet, driven by ATP hydrolysis (11). Crucially, most P4-ATPases associate with an accessory β-subunit, known as the CDC50 family (CDC50A, B, or C), to form a heteromeric complex essential for the functional maturation and trafficking of phospholipids (12). In addition, scramblases, such as TMEM16F and XKR8, mediate rapid, bidirectional lipid movement, leading to PS exposure on the cell surface during physiological events like platelet activation or apoptosis (13, 14). This study focuses on how the avian ortholog ATP11c and its CDC50A cochaperone form a functional complex to regulate PS translocation upon NDV infection to illuminate fundamental lipid-mediated viral hijacking mechanisms.

Viruses have evolved sophisticated strategies to exploit host cell lipid pathways and manipulate membrane lipid composition to their advantage (15, 16). Surface-exposed PS, often resulting from virus-induced apoptosis or direct scramblase activation, serves not only as an apoptotic signal but also is frequently hijacked by enveloped viruses (17). This strategy, termed "apoptotic mimicry," enables viruses bearing host-derived PS in their envelopes to engage PS receptors, such as TIM (T cell/transmembrane immunoglobulin and mucin) and TAM (Tyro3, AXL, Mertk) families on target cells, thereby enhancing attachment and entry (18). Beyond receptor binding, PS can directly influence membrane properties like curvature and charge or interact with viral proteins to promote critical steps, such as membrane fusion or virion budding (19). For instance, the M proteins of Ebola virus (VP40) and paramyxoviruses like respiratory syncytial virus (RSV) have been shown to directly bind PS-rich regions of the inner PM leaflet, an interaction essential for their oligomerization and efficient virus-like particle (VLP) budding (20, 21, 22).

The precise functional role of PS during distinct stages of NDV infection and the involvement of the host membrane PS transport machinery have remained unclear, particularly the ATP11C–CDC50A flippase complex. In the present study, we explored how NDV exploits host PS pathways, specifically targeting the ATP11C–CDC50A flippase complex, throughout the infection cycle. Our findings demonstrate its dual mechanism: mediating viral attachment and budding through PS interactions on virions and host membranes, and subverting host lipid asymmetry, thereby propelling infection progression. These mechanistic insights into PS-mediated pathogenesis advance understanding of NDV–host interactions.

## Results

### NDV infection induces apoptotic PS externalization to promote M protein–dependent viral budding

**Figure 1:**
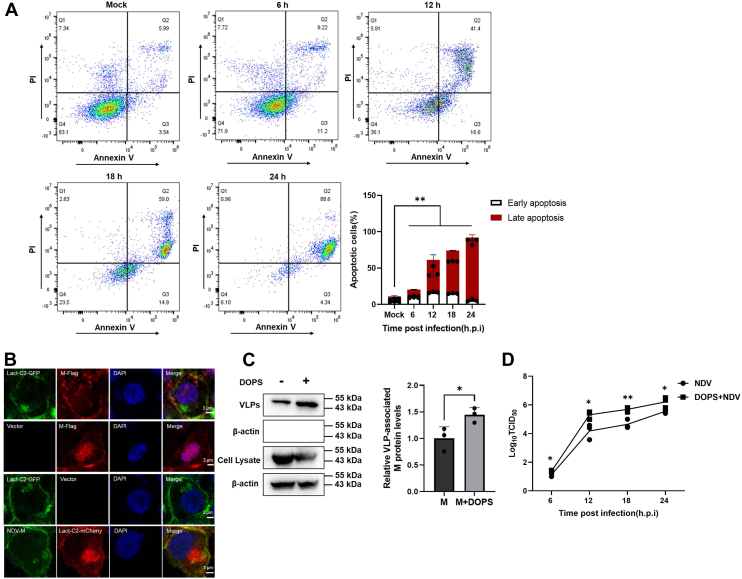
**Phosphatidylserine dynamics regulate NDV infection.***A*, kinetics of apoptosis-mediated PS exposure during NDV infection. *Top*, rrepresentative flow cytometry plots showing Annexin V/PI staining dynamics in HeLa cells infected with NDV (MOI = 1). *Bottom*, quantification of Annexin V^+^ populations over time. Mean ± SD; n = 3; Student's *t* test: 6 hpi, *p* = 0.0003; 12 hpi, *p* = 0.0001; 18 hpi, *p* = 0.0001; and 24 hpi, *p* = 0.0001. *B*, spatial coupling between NDV M protein and PS-rich membrane domains. *Upper panels,* HEK-293T cells coexpressing M-FLAG (*red*) and Lact-C2-GFP (PS probe, *green*). *Lower panels,* infected HeLa cells (12 hpi) stained with anti-M antibody (*green*) and PS-probe (Lact-C2-mCherry, *red*). Scale bars represent 5 μm. *C*, eexogenous DOPS enhances M protein–driven VLP release. *Left,* Western blot quantification of M protein in cell lysates and supernatant VLPs from exogenous DOPS (1 μM)-treated cells. β-actin: loading control. *Right*, ddensitometric analysis. Mean ± SD; n = 3; Student's *t* test: *p* = 0.0413. *D*, viral titers in supernatants of NDV-infected cells with/without DOPS supplementation (Log_10_ TCID_50_/ml). Mean ± SD; n = 3; Student’s *t* test: 6 hpi, *p* = 0.035; 12 hpi, *p* = 0.030; 18 hpi, *p* = 0.006; and 24 hpi, *p* = 0.043. MOI, multiplicity of infection.

To examine the temporal relationship between NDV infection and PS externalization, DF1 cells infected with NDV (1 multiplicity of infection) were analyzed by flow cytometry at 6-, 12-, 18-, and 24-h postinfection (hpi). Results demonstrated a progressive increase in apoptosis-associated PS exposure: early apoptosis (Annexin V^+^/propidium iodide [PI^-]^): 11.2% (6 hpi), 16.6% (12 hpi), 14.9% (18 hpi), and 4.3% (24 hpi); late apoptosis (Annexin V^+^/PI^+^): 9.2% (6 hpi), 59.0% (12 hpi), 46.6% (18 hpi), surging to 86.6% by 24 hpi. Total PS-positive cells (early + late apoptosis) increased from 20.4% (6 hpi) to 58% (12 hpi), 64.9% (18 hpi), and ultimately 90.9% at 24 hpi (*p* < 0.05 *versus* mock; Figure 1*A*). Pearson’s correlation analysis revealed a strong positive association between apoptosis progression and PS externalization (*r* = 0.92, *p* < 0.01), indicating that NDV exploits apoptosis to drive PS redistribution.

To explore the role of the NDV M protein in remodeling PS spatial dynamics, subcellular PS distribution was visualized using Lact-C2-GFP and Lact-C2-mCherry as PS-specific fluorescent probes. Confocal imaging revealed tight spatial colocalization between NDV M protein and PS at the PM during NDV infection and recombinant M-FLAG plasmid overexpression (Fig. 1*B*). These findings suggest that the viral M protein may recruit PS, a mechanism potentially critical for viral envelope anchoring and the initiation of virion assembling.

The M protein orchestrates paramyxovirus VLP assembly and budding and is sufficient to drive particle release (23, 24). Thus, M protein detection in the supernatant serves as a reliable proxy for VLP yield to a significant extent. VLP production was assessed by quantifying M protein expression in pelleted cell supernatants *via* Western blot. To assess the impact of exogenous PS on M-mediated budding, we supplemented human embryonic kidney 293T (HEK-293T) cells expressing M-FLAG with 1 μM 1,2-dioleoyl-*sn*-glycero-3-phospho-L-serine (DOPS). Western blot analysis demonstrated a 1.5-fold increase in VLP production (*p* < 0.05; Fig. 1*C*) and a concomitant 40% reduction in intracellular M protein levels in PS-treated cells compared with controls. Viral titers (tissue culture infectious dose [TCID]_50_/ml) in culture supernatants correlated with these findings, showing a 2.8 Log10 enhancement post 18 h NDV supplied with PS compared with NDV alone (*p* < 0.05; Fig. 1*D*). Together, these results indicate that PS enhances the membrane-binding affinity of the M protein that concentrates M at PS-enriched PM microdomains, thereby promoting virion assembly and release by optimizing viral envelope budding efficiency.

### Host PS governs NDV entry and replication through headgroup-specific modulation

**Figure 2:**
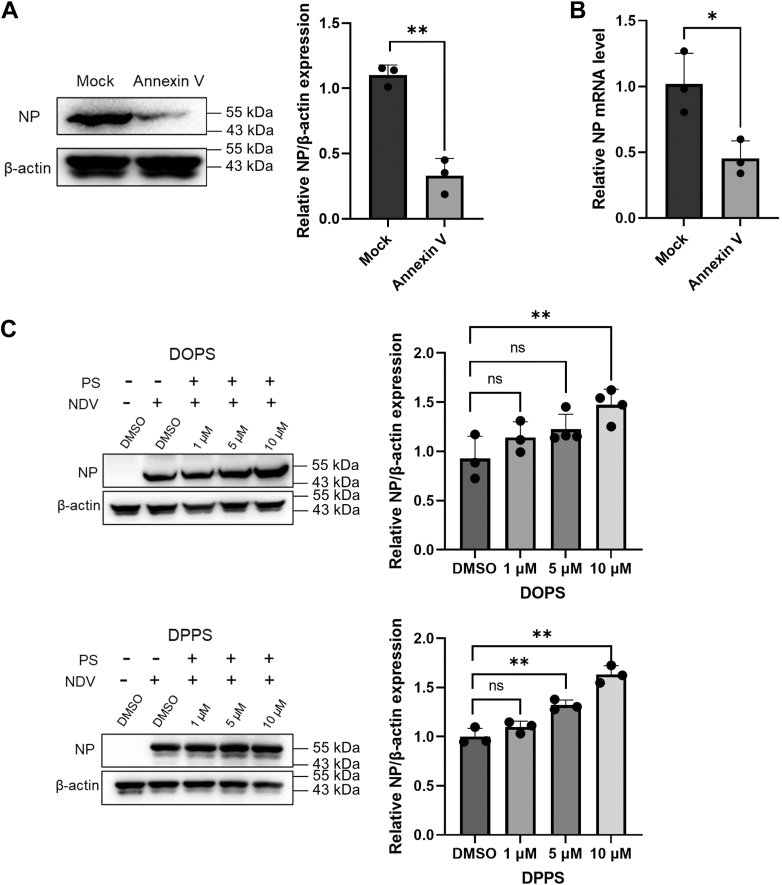
**Host PS-dependent enhancement of NDV replication.***A*, *left*, Western blot of NDV NP expression in DF-1 cells treated with apoptotic PS blocker Annexin V protein. *Right*, quantification normalized to β-actin, *p* = 0.0010. *B*, RT–qPCR analysis of NDV NP mRNA levels in Annexin V-treated *versus* control cells. Three biologically independent experiments with measurements by Student's *t* test; data are represent as mean ± SD. *p* = 0.0220. *C*, assessment of the dose–responsive effects of PS supplementation (using DOPS/DPPS, 1–10 μM) on NDV NP protein levels. Data were analyzed by one-way ANOVA for multiple comparisons *versus* control; mean ± SD; n = 3. DOPS: 1 μM, *p* = 0.3426; 5 μM, *p* = 0.1113; 10 μM, *p* = 0.0052. DPPS: 1 μM, *p* = 0.3035; 5 μM, *p* = 0.0017; 10 μM, *p* = 0.0001. DOPS, 1,2-dioleoyl-*sn*-glycero-3-phospho-l-serine; DPPS, dipalmitoyl-PS; NDV, Newcastle disease virus; NP, nucleoprotein; PS, phosphatidylserine; qPCR, quantitative PCR.

To investigate the role of PS in NDV replication, we targeted two pathways: binding virion-associated PS (*via* Annexin V protein blockade) and exogenously modulating host PS metabolism. Annexin V-mediated PS neutralization reduced both NP protein levels (by 70%) and NP mRNA abundance (by 60%) compared with untreated controls (*p* < 0.05; Fig. 2, *A* and *B*), strongly implicating PS in viral entry and early genome uncoating. In contrast, supplementation of NDV-infected DF-1 cells with exogenous PS (DOPS or dipalmitoyl-PS [DPPS]) at 1 to 10 μM enhanced NP expression in a dose-dependent manner, peaking at 1.6-fold induction at 10 μM (*p* < 0.05; Fig. 2*C*). Despite distinct acyl chains—DOPS contains monounsaturated 18:1 fatty acids, whereas DPPS comprises saturated 16:0 chains—both PS species showed comparable NP upregulation (*p* > 0.05), indicating a phosphate-serine headgroup-dependent mechanism in promoting NDV replication. These data collectively establish PS as a critical host membrane determinant regulating NDV attachment and envelope fusion.

### ATP11c facilitates NDV replication through PS asymmetry regulation

**Figure 3:**
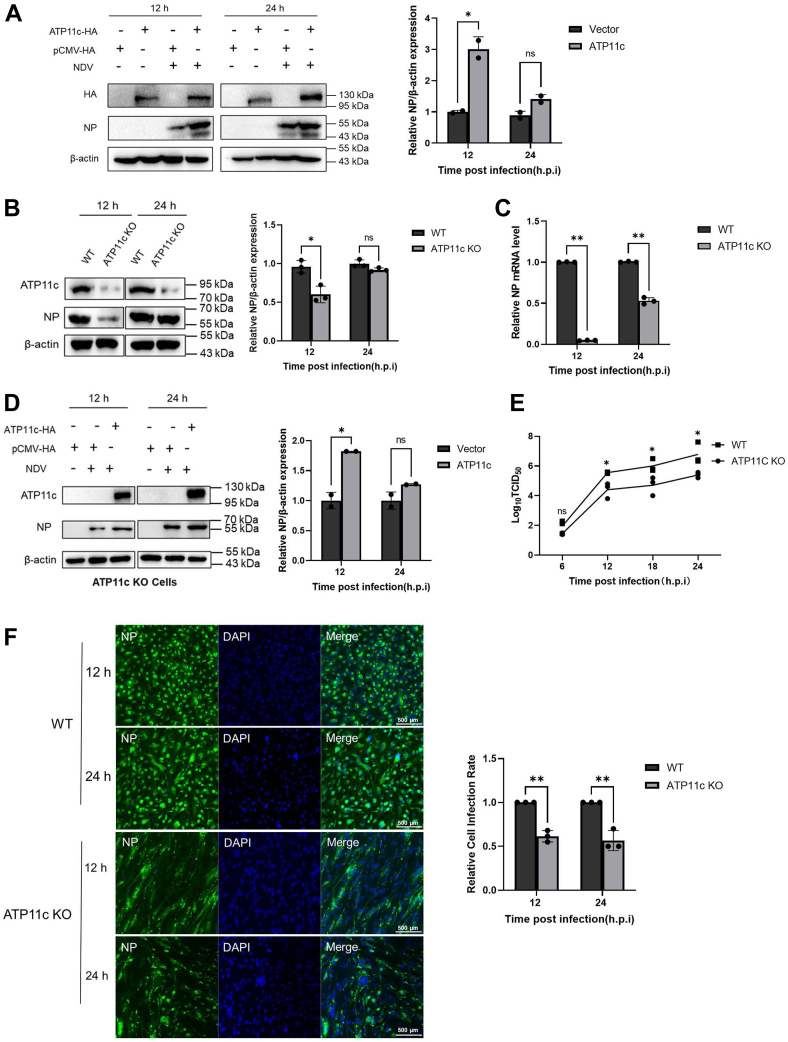
**ATP11c sustains NDV replication through maintenance of PS asymmetry.** ATP11c overexpression accelerates NDV replication kinetics. *A*, Western blot analysis of NP protein in ATP11c-expressing DF-1 cells at 12 and 24 h postinfection (hpi). *Right panel,* densitometric quantification of protein bands (normalized to β-actin), mean ± SD; n = 3; Student's *t* test: 12 hpi, *p* = 0.019; 24 hpi, *p* = 0.064. *B*, NP protein levels in WT *versus* ATP11c-KO cells. ATP11c KO impairs viral replication. Mean ± SD; n = 3; 12 hpi, *p* = 0.010; 24 hpi, *p* = 0.068. *C*, RT–qPCR of NP mRNA levels. Mean ± SD; n = 3; 12 hpi, *p* = 0.000001; 24 hpi, *p* = 0.000034. *D*, Western blot analysis of ATP11c and NP protein levels in ATP11c-KO cells expressing recombinant ATP11c. Mean ± SD; n = 3; 12 hpi, *p* = 0.013; 24 hpi, *p* = 0.116. *E*, viral titers in supernatants (Log_10_ TCID_50_/ml). Mean ± SD; n = 3; Student's *t* test: 6 hpi, *p* = 0.122; 12 hpi, *p* = 0.019; 18 hpi, *p* = 0.041; 24 hpi, *p* = 0.031. *F*, *left*, immunofluorescence detection of NP (*green*) in infected KO cells (24 hpi). Nuclei: DAPI (*blue*). Scale bar represents 20 μm. *Right*, quantification of NP-positive cells. Mean ± SD; n = 3; 6 hpi, *p* = 0.0005; 12 hpi, *p* = 0.0028. DAPI, 4′,6-diamidino-2-phenylindole; NDV, Newcastle disease virus; NP, nucleoprotein; PS, phosphatidylserine; qPCR, quantitative PCR; TCID, tissue culture infectious dose.

We next investigated whether spatial regulation of PS membrane asymmetry by ATP11c—a key phospholipid flippase that confines PS to the inner leaflet of the PM—specifically modulates later stages of the NDV lifecycle. To assess the function of ATP11c in PS flippase activity, we generated ATP11c-KO DF-1 cells using CRISPR–Cas9-mediated gene editing. To define its role in NDV replication, ATP11c-overexpressing cells (*versus* pCMV-HA control) were infected with NDV (multiplicity of infection = 1). Protein analysis revealed a 3.0-fold increase in viral NP levels at 12 hpi and a 1.3-fold elevation at 24 hpi (*p* < 0.05; Fig. 3*A*), indicating ATP11c-dependent enhancement of viral transcription and translation efficiency. These results suggest that ATP11c promotes NDV replication by maintaining PS membrane asymmetry, a critical factor for virion assembly.

In ATP11c-KO cells, NP protein levels decreased to 40% of WT levels at 12 hpi (*p* < 0.05; Fig. 3*B*), though no significant reduction was observed at 24 hpi. Conversely, NP mRNA levels in KO cells declined markedly to 10% (12 hpi) and 50% (24 hpi) of WT (*p* < 0.05; Fig. 3*C*). Complementation with ATP11c fully rescued NP expression (*p* < 0.05; Fig. 3*E*), whereas viral titers in KO supernatants showed a consistent 1-Log10 reduction from 12 to 24 hpi (Fig. 3*E*). Immunofluorescence (IF) assays further corroborated these findings: both the percentage of NP-positive cells and fluorescence intensity were significantly reduced in KO cells compared with WT (*p* < 0.05; Fig. 3*F*). Collectively, these data establish ATP11c as an essential host factor driving NDV replication, with its activity predominantly critical during early infection stages.

NDV executes a biphasic regulation of ATP11c, modulating its function and expression to facilitate infection.

**Figure 4:**
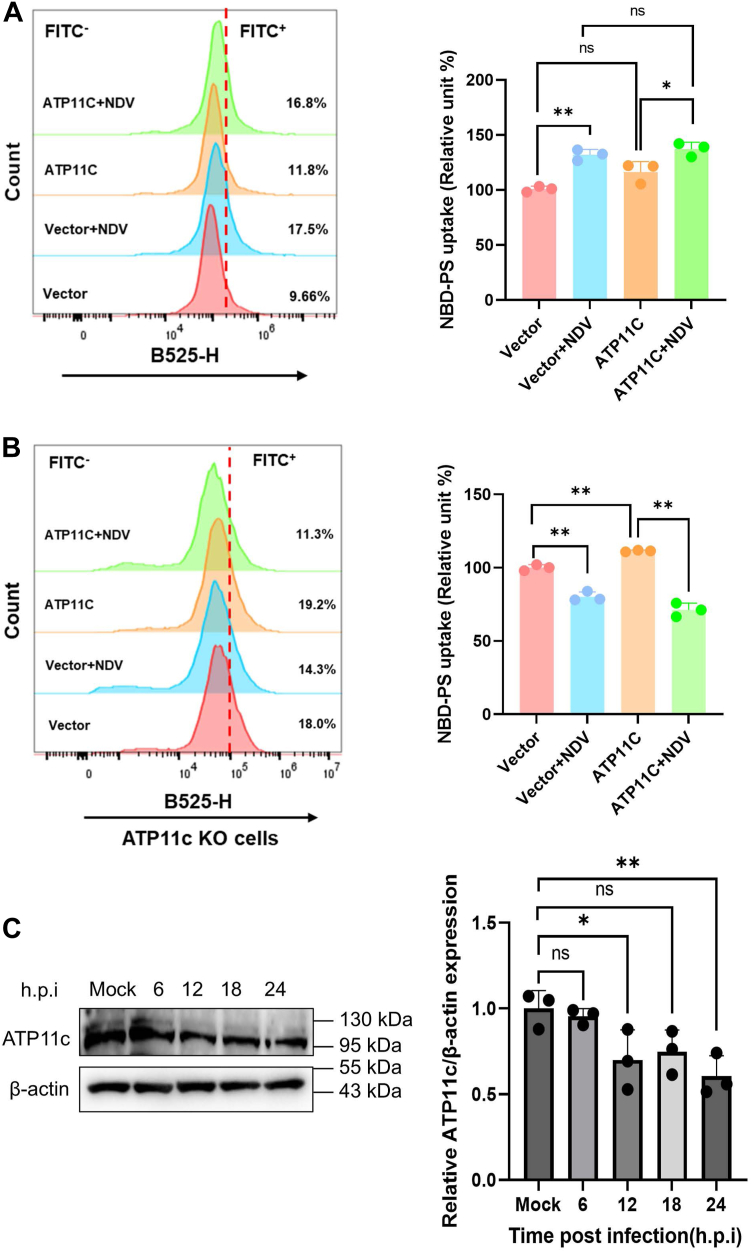
**Bidirectional subversion of ATP11c-mediated PS flipping by NDV.***A*, NDV infection enhances endogenous ATP11c flippase activity. NBD-labeled phosphatidylserine (NBD-PS) uptaking levels in ATP11c-overexpressing DF-1 cells infected with NDV. Mean ± SD; n = 3; one-way ANOVA with Tukey’s test: vector + NDV *versus* vector, *p* = 0.0006; ATP11c + NDV *versus* ATP11c, *p* = 0.0339. *B*, ATP11c KO cell rescue assay. PS flipping efficiency in ATP11c-KO cells ± complementation and NDV infection. Data normalized to WT, vector + NDV *versus* vector: *p* = 0.0009; ATP11C *versus* vector: *p* = 0.0008; ATP11C + NDV *versus* ATP11C: *p* = 0.0001. Mean ± SD; n = 3. *Dashed line*: WT baseline. *C*, NDV infection leads to a time-dependent downregulation of ATP11c protein expression. DF-1 cells were infected with NDV (MOI = 1) for the indicated durations (0, 6, 12, 18, and 24 h). Whole-cell lysates were analyzed by Western blot for the expression of endogenous ATP11c. Densitometric quantification of ATP11c protein levels, normalized to β-actin. Mean ± SD; n = 3; Student's *t* test: 12 hpi, *p* = 0.0405; 24 hpi, *p* = 0.0087. MOI, multiplicity of infection; NDV, Newcastle disease virus; PS, phosphatidylserine.

Given that ATP11c is essential for NDV replication (Fig. 3), we next investigated whether the virus, in turn, actively manipulates ATP11c′s flippase function to remodel the host membrane landscape. To test this, we first assessed PS flipping activity in ATP11c-HA-overexpressing DF-1 cells. Strikingly, at 12 hpi, NDV infection triggered a significant 35% increase in 1-oleoyl-2-{6-[(7-nitro-2-1,3-benzoxadiazol-4-yl)amino]hexanoyl}-sn-glycero-3-phosphoserine (NBD-PS) uptake compared with mock-infected controls (*p* < 0.05), suggesting that the virus initially enhances the activity of existing ATP11c to support its replication needs (Fig. 4*A*).

To further probe this dynamic, we examined the impact of NDV in ATP11c-KO cells. As expected, infection of KO cells led to a further 20% reduction in the already compromised PS uptake (Fig. 4*B*). While complementation with recombinant ATP11c restored baseline PS flipping activity, subsequent NDV infection still suppressed this restored function by 40% (*p* < 0.05; Fig. 4*B*), indicating that in later stages or under conditions of limited flippase availability, NDV actively antagonizes ATP11c function.

This apparent contradiction—enhancing activity early on but suppressing it later—led us to hypothesize that NDV employs a temporal regulatory strategy. To explore this, we monitored ATP11c protein levels over a 24-h infection time course. Western blot analysis revealed a progressive, time-dependent downregulation of endogenous ATP11c protein expression following NDV infection, with levels decreasing significantly after 12 hpi (*p* < 0.05; Fig. 4*C*).

Taken together, these data unveil a sophisticated, biphasic mechanism of viral subversion. In the early phase of infection, NDV appears to hijack and enhance the function of the existing ATP11c pool to maintain the PS asymmetry required for efficient replication, as established in Figure 3. However, as the infection progresses into its late phase, the virus orchestrates a shutdown of new ATP11c synthesis. This gradual depletion of the flippase is a strategic move that cripples the cell's ability to maintain PS asymmetry, thereby facilitating the massive PS externalization required for efficient apoptotic mimicry and virion budding, as observed in Figure 1*A*.

### Key CDC50A residues govern ATP11c-dependent PS flipping

**Figure 5:**
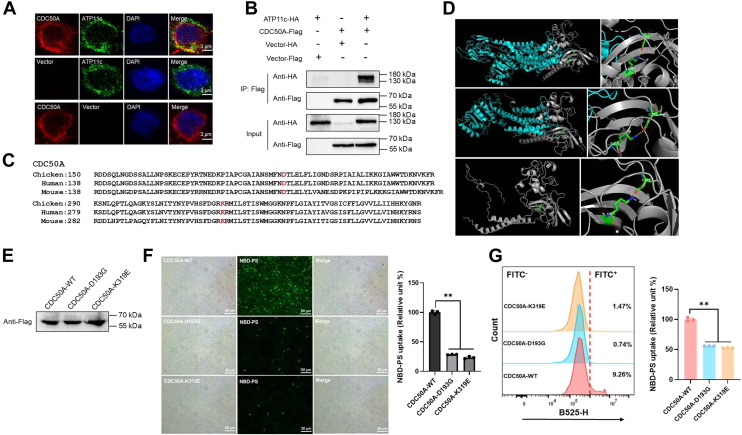
**Structural determinants of CDC50A–ATP11c functional cooperativity.***A*, IFA analysis of ATP11c- (*red*) and CDC50A-FLAG (*green*) coexpression in HEK-293T cells. *Red arrow* indicated colocalization of ATP11c and CDC50A. Scale bar represents 3 μm. *B*, co-IP validation of physical interaction. Anti-FLAG beads pulldown efficiency shown on Western blots. *C*, CDC50A protein evolutionary conservation analysis across *Gallus gallus*, human and mouse. *Red fonts*: D193 and K319 amino acid. *D*, structural models of key amino acid interactions within the ATP11C–CDC50A complex. *Top*, cryo-EM structure showing the D181–K308 interaction in the human ATP11C–CDC50A complex (Protein Data Bank ID: 7BSV). *Middle*, homology modeling of the corresponding D193–K319 interaction in the chicken complex. *Bottom*, predicted structure of chicken CDC50A (Q5F362) from the AlphaFold Protein Structure Database. Structures were visualized in PyMOL (C: *green*, N: *blue*, O: *red*), and potential ionic/hydrogen bonds are depicted as *yellow dashes*. *E*, Western blot confirming equivalent expression of CDC50A variants. *F*, NBD-PS internalization assay demonstrating loss-of-function CDC50A mutants (D193G/K319E) *versus* WT, normalized to CDC50A-WT, D193G: *p* = 0.0001; K319E: *p* = 0.0001. Scale bars represent 50 μm. *G*, flow cytometry quantification of PS uptake flipping efficiency. MMean ± SD; n = 3; Student's *t* test. CDC50A-WT *versus* D193G: *p* = 0.0001; CDC50A-WT *versus* K319E: *p* = 0.0001. co-IP, coimmunoprecipitation; HEK-293T, human embryonic kidney 293T cell line; IFA, immunofluorescence assay; PS, phosphatidylserine.

To analyze the mechanistic interplay between ATP11c and its obligate subunit CDC50A, we first interrogated their complex formation. Coexpression of ATP11c-HA and CDC50A-FLAG in HEK-293T cells revealed marked PM colocalization by IF (Fig. 5*A*), contrasting with cytoplasmic or membrane-only localization when expressed individually. Coimmunoprecipitation (co-IP) assays confirmed a direct physical interaction, with FLAG-tagged CDC50A pulldown enriching ATP11c-HA (Fig. 5*B*), supporting their assembly into a functional phospholipid-translocating complex.

We next defined the structural determinants of CDC50A critical for ATP11c activity through mutagenesis strategy. Residues Asp193 (D193) and Lys319 (K319) within the cytoplasmic domain of CDC50A were chosen for site-directed mutagenesis based on their high degree of conservation across multiple species (Fig. 5*C*), their predicted location within functional cytoplasmic domains potentially involved in protein interactions or modulation of ATP11c activity; and the presence of charged residues (Asp/Lys), making them prime candidates for electrostatic interactions critical for complex function. Comparative structural analysis revealed that D193 and K319 constitute an evolutionarily conserved functional motif in CDC50A. Examination of the human ortholog (Protein Data Bank ID: 7BSV) demonstrated analogous residues (D181/K308) form critical intramolecular contacts crucial for structural integrity. Homology modeling confirmed spatial positional conservation in chicken CDC50A, consistent with AlphaFold predictions (Fig. 5*D*). In addition, site-specific mutation plasmids (D193G, K319E) did not affect protein expression but severely compromised ATP11c flippase potency (Fig. 5*E*). Fluorescence NBD labeling PS uptake rate showed approximately 60% reduction in by CDC50A mutants *versus* WT CDC50A (*p* < 0.05; Fig. 5*F*). Furthermore, flow cytometry corroborated these findings, CDC50A mutants retained only about 50% of CDC50A-WT activity (*p* < 0.05; Fig. 5*G*). These data establish D193 and K319 as pivotal residues that drive CDC50A’s allosteric regulation of ATP11c, enabling efficient phospholipid flipping and homeostasis.

### Synergistic control of PS asymmetry *via* ATP11c–CDC50A structural cooperativity

**Figure 6:**
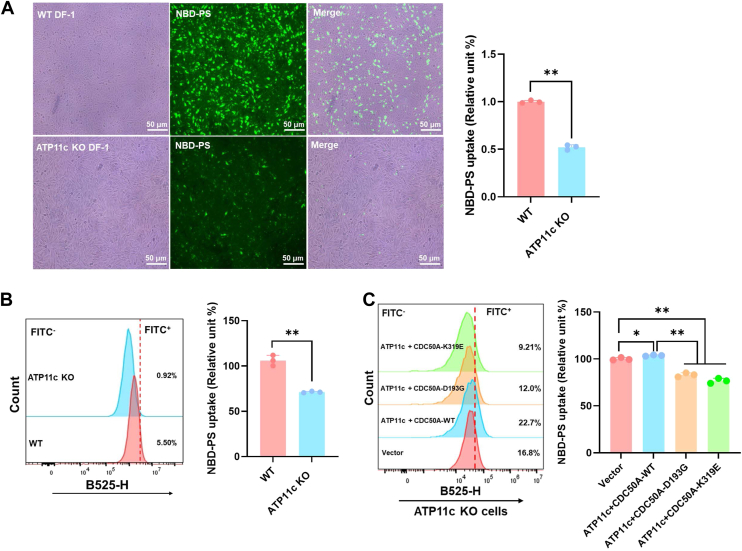
**Codependence of ATP11c–CDC50A in restoring PS membrane asymmetry.** ATP11c KO disrupts PS homeostasis. *A*, representative images of NBD-PS distribution. Scale bars represent 50 μm. NBD-PS distribution quantified by fluorescence microscopy. Mean ± SD; n = 3; Student's *t* test, *p* = 0.0001. *B*, flow cytometry analysis of NBD-PS internalization efficiency in ATP11c KO and WT cells. Mean ± SD; n = 3; Student's *t* test, *p* = 0.0005. *C*, rescue efficiency suppression by CDC50A mutant variants. ATP11c-complemented KO cells cotransfected with WT or mutant CDC50A. Mean ± SD; n = 3; one-way ANOVA with Tukey’s test, ATP11c + CDC50A-WT *versus* vector: *p* = 0.0143; ATP11c + CDC50A–D193G *versus* vector: *p* = 0.0003; ATP11c + CDC50A–K319E *versus* vector: *p* = 0.0002; ATP11c + CDC50A–D193G *versus* ATP11c + CDC50A-WT: *p* = 0.0001; ATP11c + CDC50A–K319E *versus* ATP11c + CDC50A-WT: *p* = 0.0001. PS, phosphatidylserine.

To further investigate the functional synergy between ATP11c and CDC50A in remodeling PS membrane asymmetry, we employed ATP11c KO cell lines. Genetic ablation of ATP11c markedly disrupted PS homeostasis, evidenced by 54% lower NBD-PS uptake (*p* < 0.05; fluorescence microscopy, Fig. 6*A*) and 40% reduced PS internalization (*p* < 0.05; flow cytometry, Fig. 6*B*) compared with WT controls. Functional rescue experiments further solidified their codependent relationship: while reintroduction of ATP11c reversed the PS flipping defect, coexpression of CDC50A-WT or mutants counteracted this rescue in a mutation-selective manner. Notably, the CDC50A mutants—carrying interfacial residue substitutions—exhibited dominant-negative effects, suppressing ATP11c rescue efficiency by 20% *versus* WT CDC50A (*p* < 0.05; Fig. 6*C*). These findings collectively establish that ATP11c and CDC50A form a structural and functional complex, wherein CDC50A’s conserved D193/K319 residues act as allosteric modulators to fine-tune ATP11c-driven PS flipping.

## Discussion

The complex interplay between viruses and host cell lipid metabolism is increasingly recognized as a critical determinant of infection outcomes, profoundly influencing viral entry, replication, assembly, and egress (4, 25). This study provides compelling evidence that NDV actively manipulates host PS dynamics, specifically targeting the regulatory function of the ATP11c–CDC50A flippase complex, to facilitate key steps in its life cycle. Our findings uniquely illuminate how NDV subverts cellular phospholipid transport, expanding the known repertoire of viral strategies that hijack host lipid machinery (26, 27).

Our initial findings underscore the pivotal role of PS throughout the NDV infectious cycle. The observation that NDV infection triggers substantial PS externalization on the host cell PM, correlating with apoptosis progression, aligns with mechanisms employed by numerous viruses (28). Exposed PS, either on the host cell surface or incorporated into the viral envelope during budding, facilitates viral attachment and entry by engaging PS receptors or mediating direct electrostatic interactions that promote membrane fusion (18, 29). Our results, demonstrating that masking virion surface PS with Annexin V protein significantly curtailed NDV infectivity and NP expression, strongly implicate virion-associated PS in mediating early infection events, likely encompassing both attachment and membrane fusion steps. Furthermore, the dose-dependent enhancement of NDV replication upon exogenous PS supplementation indicates that PS availability is also crucial, possibly by optimizing membrane microenvironments for replication complex assembly or enhancing viral budding efficiency.

As the principal orchestrator of paramyxovirus assembly and budding, the M protein bridges the viral ribonucleoprotein core with the host-derived lipid envelope (30). M proteins frequently possess basic residues facilitating electrostatic interactions with anionic lipids like PS, anchoring them to the inner PM leaflet (31). Our demonstration that exogenous PS significantly boosts M protein-mediated VLP production and progeny virus release provides strong evidence that direct M protein engagement with PS-rich microdomains is a critical driver for NDV assembly and budding. This mechanism mirrors findings for viruses like Ebola, where VP40's interaction with inner leaflet PS is essential for oligomerization and efficient budding (20), and RSV, whose M protein also selectively targets and clusters PS (22). This selective lipid interaction likely promotes the necessary membrane curvature for virion formation. In addition, PS externalization is a conserved strategy among enveloped viruses, molecular mechanisms governing spatial lipid control differ markedly. RSV employs its M protein to selectively cluster PS through electrostatic interactions, a process necessary for viral particle assembly. Similarly, NDV leverages membrane-bound PS to promote M protein oligomerization. Furthermore, our findings reveal a unique dependence on ATP11c–CDC50A-mediated inner-leaflet PS asymmetry, contrasting with RSV’s reliance on scramblase-independent mechanisms. In contrast, Ebola virus (EBOV) directly recruits TMEM16F, a calcium-activated scramblase, to license PS externalization during budding, circumventing host flippase regulation. Notably, recent work by Amiar *et al*. (21) illustrates that EBOV’s VP40 protein further requires a densely packed, fatty acid–ordered membrane environment to nucleate budding, whereas NDV actively employed flippase machinery to manipulate PS topology. These contrasts underscore the evolutionary plasticity of viral lipid exploitation: while EBOV prioritizes scramblase activation for “lipid scrambling”, NDV coopts flippase complexes to sustain transient asymmetry critical for M protein anchoring, suggesting clade-specific adaptations rooted in host membrane biophysics.

This study moves beyond the general role of PS to dissect the involvement of the specific machinery maintaining its asymmetric distribution—the ATP11c–CDC50A flippase complex. We establish, for the first time in the context of NDV, that ATP11c functions as a crucial host factor. ATP11c overexpression significantly accelerated viral replication kinetics, whereas CRISPR–Cas9-mediated KO profoundly impaired viral gene expression, infectious particle production, and overall infection spread. This dependence highlights the necessity of maintaining regulated PS asymmetry, particularly its concentration on the inner leaflet, for optimal NDV replication. This controlled environment is likely required for efficient M protein recruitment and oligomerization at budding sites or potentially to prevent premature host responses triggered by aberrant PS exposure before virion assembly culminates.

Intriguingly, our data suggest a dynamic, potentially bidirectional interplay between NDV and the ATP11c flippase. While baseline ATP11c activity supports infection, NDV infection appeared to modulate flippase function. In ATP11c-KO cells, infection further suppressed the residual PS flipping activity, indicating that in the absence of its primary target, NDV might antagonize other compensatory mechanisms or directly interfere with remaining flippase function. This complex manipulation underscores a sophisticated viral strategy to co-opt host lipid transport for its benefit, shifting membrane dynamics as the infection progresses.

Beyond hijacking host lipid asymmetry, NDV may further exploit apoptotic pathways to disable host antiviral responses. Our study demonstrated that NDV infection triggers robust apoptosis, correlating with severe PS externalization and M protein–mediated virion budding. Notably, while ATP11c–CDC50A functions as a guardian to restrain PS exposure, caspase-3—the executioner protease of apoptosis—has been proven to cleave P4-ATPases at a conserved N-terminal motif, thereby inactivating their flippase activity (32). We speculate that caspase-3-mediated ATP11c cleavage at late infection stages (>18 h.p.i) may disrupt PS asymmetry, shifting the balance toward scramblase-driven PS externalization. This self-amplifying loop not only sustains viral budding but also facilitates apoptotic mimicry for secondary infection.

Furthermore, our investigation confirms the indispensable functional partnership between ATP11c and its chaperone, CDC50A. Their physical interaction and colocalization at the PM are prerequisites for efficient PS transport. Critically, we demonstrated two highly conserved residues within the CDC50A domain, D193 and K319, as pivotal for conferring full flippase activity upon ATP11c. Mutation of these residues drastically reduced PS transport efficiency, mirroring the ATP11c loss-of-function phenotype and aligning with studies highlighting the importance of the CDC50A ectodomain structure for P4-ATPase function and substrate interaction (33). Our structural analysis substantiates the functional prioritization of D193–K319: They constitute a spatially conserved motif critical for CDC50A structural integrity, independent of sequence conservation alone. This intramolecular stabilization mechanism is essential for CDC50A's capacity to chaperone ATP11c, explaining the profound (>60%) flipping deficiency upon mutagenesis. The dominant-negative effect observed when expressing these CDC50A mutants during ATP11c rescue experiments in KO cells unequivocally demonstrates their functional interdependence and reinforces the notion that NDV hijacks the entire functional ATP11c–CDC50A complex, not merely ATP11c in isolation.

**Figure 7:**
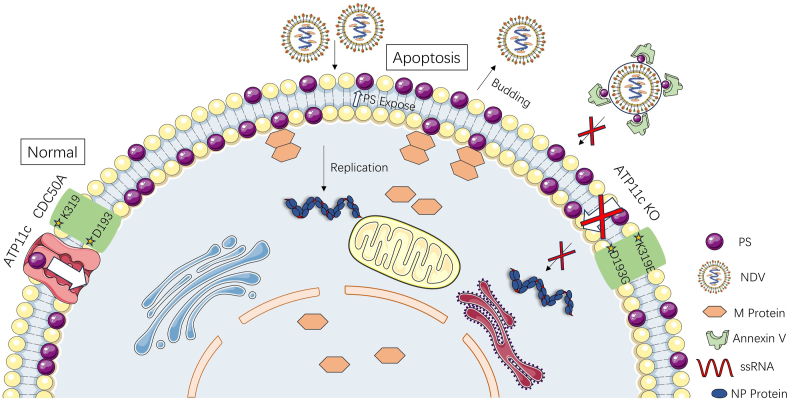
**Schematic diagram for NDV exploitation of ATP11c–CDC50A-mediated PS asymmetry to facilitate viral replication cycle.** The ATP11c–CDC50A complex maintains inner-leaflet PS asymmetry, enabling NDV M protein oligomerization at PS-rich domains to drive membrane curvature and virion assembly. In ATP11c-KO or CDC50A-mutant (D193G/K319E) cells, disrupted flippase activity reduces PS flipping efficiency, impairing M recruitment and viral budding. *Dashed lines* indicate mechanistic disruptions observed in functional assays. Colocalization of M with PS and reduced viral titers in KO models highlight the pathway’s necessity for NDV replication. M, M protein; NDV, Newcastle disease virus; PS, phosphatidylserine.

We propose a model wherein NDV strategically exploits this host lipid transport system: initially leveraging or even transiently enhancing ATP11c–CDC50A activity to ensure sufficient inner leaflet PS for M protein recruitment and optimal budding platform formation, while later contributing to the PS externalization associated with apoptosis, which may facilitate entry into new cells *via* apoptotic mimicry. This work moves beyond describing NDV's reliance on host lipids to revealing its active subversion of specific lipid transport pathways. While further research is warranted to delineate the precise molecular interactions between the M protein and PS, the mechanisms by which NDV modulates ATP11c–CDC50A activity throughout infection, and potential interplay with other lipid regulators, our findings strongly pinpoint the ATP11c–CDC50A complex as a key node in NDV–host interactions (Fig. 7).

In conclusion, this study significantly advances our understanding of NDV pathogenesis by elucidating a novel mechanism of host manipulation centered on PS dynamics and the ATP11c–CDC50A flippase complex. Targeting this host flippase complex represents a promising, previously unexplored avenue for developing novel antiviral strategies aimed at disrupting viral assembly and release.

## Experimental procedures

### Materials

HEK-293T and DF-1 cell lines (American Type Culture Collection) were maintained in our laboratory under standard conditions. The cell lines used in this study underwent commercially certified short tandem repeat profiling to authenticate their identity and mycoplasma testing to confirm the absence of contamination. All cells were cultured in Dulbecco's modified Eagle's medium (DMEM) supplemented with 10% fetal bovine serum (FBS) with routine passaging, and cell lines were free from mycoplasma contamination. DF1 chicken embryonic fibroblasts were chosen for infection studies as they model the natural avian host environment and are highly susceptible to NDV. HEK-293T cells were selected for transient overexpression, colocalization imaging (IF), co-IP, and VLP production experiments because of their high transfection efficiency and robust recombinant protein expression capabilities. The NDV vaccine strain Mukteswar was propagated in specific pathogen-free chicken embryos. Viral stocks were aliquoted and stored at −80 °C prior to experimental use.

### Plasmids, reagents, and antibodies

Opti-MEM I reduced serum medium (Thermo Fisher Scientific; catalog no.: 31985070), DMEM (Gibco; catalog no.: C11995500BT), and FBS (ExCell Biotechnology; catalog no.: FSP500). The molecular toolkit included SYBR Green quantitative PCR (qPCR) Mix (Dongsheng Biotech; catalog no.: P2092c), polyethyleneimine MW40000 (Nonin Biological Technology; catalog no.: NBS4000), and phospholipids: 18:1 PS (DOPS; Sigma–Aldrich; catalog no.: 840035P) and 16:0 PS (DPPS; Sigma–Aldrich; catalog no.: 840037P). Antibodies featured anti-FLAG (MBL Beijing Biotechnology; catalog no.: M185-3L), anti-β-actin (Sigma–Aldrich; catalog no.: SAB5600204), anti-ATP11c (Antibodies.com; catalog no.: A88371), and secondary antibodies (Thermo Fisher Scientific; Alexa Fluor 594/488; Cell Signaling Technology; horseradish peroxidase linked). NDV NP- and M-specific mouse monoclonal antibodies generated in-house. Key plasmids (Addgene; lentiCRISPRv2 #52961, psPAX2 #12260, pMD2.G #12259) and enzymatic tools (Phanta Max polymerase; Vazyme Biotechnology; P505; mutagenesis kits: C214) completed the experimental setup. NBD-PS (Avanti cat. no. 810194C) was used as 5 μM in serum-free DMEM.

### Construction of ATP11c and CDC50A recombinant plasmids

ATP11c (GenBank: 422254) and CDC50A (GenBank: 421861) were amplified from *Gallus gallus* DF-1 cell complementary DNA using Phanta Max Super-Fidelity DNA polymerase (Vazyme). Primers (synthesized by Sangon Biotech) were designed as follows: ATP11c-HA: Forward (F): 5′-GGAATTCCAGCCACCATGCTCCGCCGCAGC (EcoRI), Reverse (R: 5′-GGGGTACCTTAAAGTATCCTGAGAGGATATCTTTCTGCCAAAT (KpnI); CDC50A-FLAG: F: 5′-GGAATTCGCCACCATGGCGGTCAACTAC (EcoRI), R: 5′-GCTCTAGAATTGGGAATGTCTGCACTAGTGTTTCT (XbaI). PCR conditions: 95 °C for 3 min; 35 cycles of 95 °C 15 s, 60 °C 15 s, 72 °C 2 min; final extension at 72 °C for 5 min. Gel-purified products were digested with EcoRI/KpnI (ATP11c) or EcoRI/XbaI (CDC50A), ligated into pCMV-HA or p3×FLAG-CMV14 vectors (T4 ligase, 16 °C, 8 h), transformed into *Escherichia coli*, and selected in ampicillin-LB medium. Plasmids (ATP11c- and CDC50A-FLAG) were validated by sequencing (Sangon Biotech). For CDC50A point mutants (D193G and K319E), site-directed mutagenesis was performed using the CDC50A-FLAG plasmid as template. The following primers (designed by Vazyme Biotech and synthesized by Sangon Biotech) were used: CDC50A-D193G: F: 5′-GTTTAATGGTACATTGGAATTATACCACATTGAAAAC, R: 5′-CCAATGTACCATTAAACATACTGTTGGCAATAGCCC; CDC50A-K319E: F: 5′-TGGACGAGAAAGAATGATCCTAAGCACAATCTCATG, R: 5′-TCATTCTTTCTCGTCCATCAAAACTGTGTACAGG. Mutagenesis PCR conditions: 95 °C for 30 s; 35 cycles of 95 °C 15 s, 60 °C 15 s, 72 °C 2 min; final extension at 72 °C for 5 min. DpnI (1 μl, 37 °C, 1 h)-digested products were subjected to homologous recombination (Exnase II, 20 μl reaction: 4 μl product, 4 μl 5 × CE II Buffer, 2 μl Exnase II, 10 μl H_2_O), transformed into competent cells, and sequenced. Validated mutants (CDC50A–D193G and CDC50A–K319E) were extracted for downstream applications.

### Structural validation of CDC50A residues

The human ATP11c–CDC50A complex cryo-EM structure (Protein Data Bank ID: 7BSV) served as the template for predicting the structure of the chicken ortholog using SWISS-MODEL homology modeling, with specific focus on mapping the spatial positions and potential interactions of residues D193 and K319 in chicken CDC50A (corresponding to D181 and K308 in the human CDC50A template); for validation, we additionally retrieved the AlphaFold-predicted structure of chicken CDC50A (UniProt ID: Q5F362, AF-Q5F362-F1) from the AlphaFold Protein Structure Database to independently assess the structural environment of residues D193 and K319, comparing β-sheet topologies and analyzing spatial proximity and electrostatic compatibility between their side chains using PyMOL (Schrodinger, Inc), version 2.4.0, with atomic representations (C: *green*, N: *blue*, and O: *red*) and potential ionic/hydrogen-bonding interactions depicted as *yellow dashes*.

### Generation of ATP11c KO cell lines

To generate ATP11c KO cell lines, two single-guide RNAs (sgRNAs) targeting *Gallus gallus* ATP11c were designed using the Synthego CRISPR tool and synthesized (Sangon Biotech) with the following sequences: sgRNA-1: F: 5′-caccgTCATTGCCCCAACAGTGTGC (BsmBI overhang), R: 5′-aaacGCACACTGTTGGGGCAATGAc (BsmBI overhang); sgRNA-2: F: 5′-caccgCATTGCCCCAACAGTGTGCC, R: 5′-aaacGGCACACTGTTGGGGCAATGc.

Annealed sgRNA oligo duplexes were ligated into BsmBI-linearized lentiCRISPRv2 vector. The recombinant plasmids (ATP11c-sgRNA-1/2) were verified by sequencing. For lentivirus production, HEK-293T cells were cotransfected with 6 μg recombinant plasmid, 2 μg pMD2.G, and 8 μg psPAX2 using polyethyleneimine. Viral supernatant was collected 48 h post-transfection, filtered, and stored at −80 °C. Prior to infection, puromycin sensitivity in DF-1 cells was determined by dose–response assays (1:5000 dilutions; minimal lethal concentration selected). Lentivirus-infected DF-1 cells underwent puromycin selection (3 days), followed by single-cell cloning in 96-well plates. Clonal lines were validated by PCR: Genomic DNA was amplified with primers flanking sgRNA target sites: F: 5′-TCATTAAGCTTGGAAAAGACCACTGAGAT (HindIII site), R: 5′-CCTGACCTGCCCTTTCCCCAA. PCR products were cloned into pMD-19T vector and sequenced. Western blot: Total protein lysates (radioimmunoprecipitation assay buffer) were probed with anti-ATP11c antibody (Santa Cruz; sc-123; 1:1000 dilution) to confirm depletion.

### IF assay

Cells grown on coverslips in 6-well plates were fixed with 4% paraformaldehyde for 15 min at room temperature and permeabilized with 0.5% Triton X-100 for 10 min. After blocking with 3% bovine serum albumin in PBS at 37°C for 1 h, cells were incubated with primary antibodies diluted in PBS overnight at 4°C. Following three washes with PBS containing 0.1% Tween-20, cells were stained with fluorescence-conjugated secondary antibodies (*e.g.*, Alexa Fluor 488/594) for 1 h at room temperature, protected from light. After additional PBS containing 0.1% Tween-20 washes, coverslips were mounted on glass slides using antifade mounting medium containing 4′,6-diamidino-2-phenylindole. Images were acquired using a Zeiss LSM 880 confocal laser scanning microscope (Carl Zeiss AG) under 63× oil immersion objectives.

### qRT–PCR

Total RNA was isolated from cultured cells using TRIzol Reagent (Thermo Fisher Scientific), followed by reverse transcription into complementary DNA with PrimeScript RT Reagent Kit (Takara Bio). Real-time qPCR amplification was performed on a CFX Connect Real-Time PCR Detection System (Bio-Rad) using SYBR Green Master Mix with the following gene-specific primers: NDV NP: F: 5′-CAGGGTATCGGTGATGTCTTCT-3′, R: 5′-CAACAATAGGAGTGGAGTGTCTGA-3′; β-actin (internal control): F: 5′-TATTGCTGCGCTCGTTGTTGAC-3′, R: 5′-GATACCTCTTTTGCTCTGGGCTTC-3′. Thermal cycling included initial denaturation (95 °C, 30 s), 40 cycles of 95 °C for 5 s and 60 °C for 30 s, with melt curve analysis (65–95 °C) to confirm specificity. Relative mRNA expression was calculated by the 2−ΔΔCT method normalized to β-actin, and the data were analyzed using Bio-Rad CFX Maestro Software.

### Flow cytometric apoptosis analysis

Cells were detached with 0.25% trypsin–EDTA (Thermo Fisher Scientific), and digestion was quenched using DMEM containing 10% FBS (Gibco). The cell suspension was pelleted by centrifugation at 200*g* for 5 min, followed by two washes with PBS (HyClone). Cell density was adjusted to 1 × 10^6^ cells/ml in Annexin V Binding Buffer (BD Biosciences), and viability (>95%) was confirmed by trypan blue exclusion assay (Countess II) prior to staining. Cells were dual-stained with 5 μl Annexin V-FITC (1:40 dilution) and 10 μl PI (20 μg/ml; Sigma–Aldrich) for 20 min at 25 °C in the dark. After staining, samples were protected from light on ice and analyzed within 1 h using a CytoFLEX Flow Cytometer (Beckman Coulter) equipped with 488 nm and 561 nm lasers. Gating was based on forward/side scatter to exclude debris and doublets, and apoptotic populations were quantified *via* CytExpert Software (version 2.3) with unstained and single-stained controls for compensation. Quantitative data from three independent biological replicates are presented as mean ± SD.

### Viral titer determination

Viral titers were quantified *via* the 50% TCID_50_ endpoint dilution assay. DF1 cells were seeded in flat-bottom 96-well plates and cultured until reaching 90 to 95% confluency (37 °C, 5% CO_2_). Supernatants from infected cells were serially diluted in DMEM (10-fold increments from 10^−1^ to 10^−11^), and 100 μl of each dilution was inoculated in triplicate into designated columns of the preseeded plates (one dilution per column). The terminal column of each plate was reserved for negative controls (DMEM only). Following 72 h of incubation under standard culture conditions, cytopathic effects, including cell rounding, detachment, and lysis, were recorded by light microscopy (Olympus IX73; 200× magnification). Viral titers (TCID_50_/ml) were calculated using the Reed–Muench formula with the following criteria: wells exhibiting >50% cytopathic effect–positive cells were scored as infected.

### Co-IP assay

HEK-293T cells were transfected in 6 cm dishes using Lipofectamine 2000 (Thermo Fisher, catalog no.: 11668019) per the manufacturer’s protocol (DNA:Lipo 2000 ratio = 1:2). After 36 h, cells were lysed in ice-cold radioimmunoprecipitation assay buffer (25 mM Tris–HCl [pH 7.6], 150 mM NaCl, 1% NP-40, 1% sodium deoxycholate, and 0.1% SDS) with 1× protease inhibitors. Lysates were centrifuged (12,000*g*, 15 min, 4 °C), and supernatants were collected. Dynabeads Protein G (20 μl; Thermo Fisher) were conjugated with 2 μl anti-FLAG antibody (Sigma–Aldrich, F3165) in Tris-buffered salien with Tween-20 (30 min, 25 °C), washed, and then incubated with lysates overnight at 4 °C. Beads were washed with Tris-buffered saline with Tween-20, and proteins were eluted in SDS loading buffer. Input controls (10% lysate) and IP samples were boiled (95 °C, 5 min) and for Western blot analysis.

### Data analysis

Statistical analyses of qRT–PCR results, protein expression levels, viral titers, and flow cytometry data were performed using Student's *t* test or one-way ANOVA with Bonferroni correction for multiple comparisons. All statistical calculations and graphical representations were generated using Prism 8 software (GraphPad Software).

## Data availability

The data that support the findings of this study are available from the corresponding author upon reasonable request.

## Supporting information

This article contains supporting information.

## Conflict of interest

The authors declare that they have no conflicts of interest with the contents of this article.
