# Supplementary material for: Newcastle disease virus exploits the phospholipid flippase ATP11c–CDC50A complex to promote viral infection
Source: J Biol Chem. 2025 Aug 12;301(9):110584. doi: 10.1016/j.jbc.2025.110584 (PMC12446626; doi:10.1016/j.jbc.2025.110584)
Supplement: Supplementary data [file mmc1.docx]

**Supplementary Experimental procedures**

**Materials and Methods**

**Cell Culture and NDV Infection**

DF-1 cells were cultured in Dulbecco's Modified Eagle Medium (DMEM) supplemented with 10% fetal bovine serum (FBS). For the time-course analysis, cells were seeded in 6-well plates and grown to 80-90% confluency. The cells were then infected with NDV at a multiplicity of infection (MOI) of 1. A mock-infected group was treated with an equal volume of allantoic fluid and served as the 0-hour control. Cells were harvested at 0, 6, 12, 18, and 24 hours post-infection (hpi).

**Western Blot Analysis**

Total protein was extracted from harvested cells using RIPA lysis buffer containing a protease inhibitor cocktail. Protein concentration was determined using a BCA Protein Assay Kit. Equal amounts of protein (30 µg) per sample were separated by 10% SDS-PAGE and subsequently transferred to a polyvinylidene fluoride (PVDF) membrane. The membranes were blocked with 5% non-fat milk in Tris-buffered saline with 0.1% Tween 20 (TBST) for 1 hour at room temperature.

Membranes were then incubated overnight at 4°C with the following primary antibodies: anti-TMEM16F (Thermo Fisher, PA5-88322), anti-XKR8 (CUSABIO, PA875681LA01HU), anti-ABCA3 (Thermo Fisher, PA5-103632), and anti-β-actin. After washing three times with TBST, the membranes were incubated with HRP-conjugated secondary antibodies for 1 hour at room temperature. Immunoreactive bands were visualized using an enhanced chemiluminescence (ECL) detection system. Densitometric analysis was performed using ImageJ software (NIH), and the intensity of each target protein band was normalized to its corresponding β-actin band.

**Supplementary Results**

**NDV Infection Suppresses the Expression of Host Scramblase and Floppase**

To investigate whether NDV infection broadly impacts the cellular machinery that governs phospholipid asymmetry, we assessed the expression of key transporters over a 24-hour infection period. As shown in Supplementary Figure S1, Western blot analysis revealed a progressive and significant decrease in the protein levels of the scramblases TMEM16F and XKR8 as the infection advanced. Similarly, the expression of the floppase ABCA3 was also markedly reduced in a time-dependent manner. Collectively, these data indicate that NDV infection orchestrates a widespread suppression of host phospholipid transporters, a mechanism that likely facilitates the viral life cycle by disrupting plasma membrane homeostasis.

**Supplementary Figure Legend**


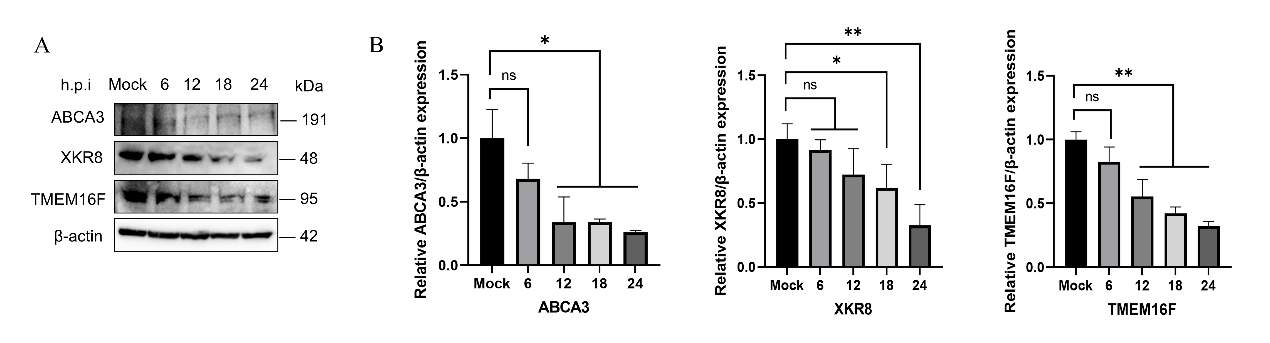


Figure S1. NDV infection leads to a time-dependent downregulation of host phospholipid transporters. DF-1 cells were mock-infected (0h) or infected with NDV for the indicated durations (6, 12, 18, and 24h). Whole-cell lysates were analyzed by Western blot for the expression of scramblases (TMEM16F, XKR8) and a floppase (ABCA3). β-actin was used as an internal loading control. (A) Representative immunoblots from one of three independent experiments. (B) Densitometric quantification of protein levels. The band intensities of target proteins were normalized to β-actin. Data are presented as the mean ± SD from three independent experiments. Statistical significance was calculated by comparing each time point to the 0h control. ABCA3: 6h: p=0.0990:12h: p=0.0448:18h: p=0.0298:24h: p=0.0220. XKR8: 6h: p=0.3655: 12h: p=0.1119:18h：p=0.0407: 24h：p=0.0044. TMEM16F: 6h: p=0.0818: 12h: p=0.0063: 18h: p=0.0002:24h：p=0.0001 (*p < 0.05; **p < 0.01).
